# The Overdiagnosis of Thyroid Micropapillary Carcinoma: The Rising Incidence, Inert Biological Behavior, and Countermeasures

**DOI:** 10.1155/2021/5544232

**Published:** 2021-07-09

**Authors:** Shujian Xu, Yong Han

**Affiliations:** Department of Thyroid and Breast Surgery, Binzhou Medical University Hospital, No. 661, Huangheer Road, Bincheng District, Binzhou 256603, China

## Abstract

The incidence of papillary thyroid microcarcinoma (PTMC) has exponentially increased in the past three decades. The 2014 World Cancer Report stated that, among the new cases of thyroid carcinoma, >50% are PTMC. The incidence of thyroid cancer was growing by 20.1% annually in China. Most of PTMC have low risk and excellent prognoses. It must be realized that the problem of overdiagnosis of thyroid cancer is quite serious. In this article, we summarized the phenomenon and the cause of “overdiagnosis” of PTMC, the progress of clinical management, and the countermeasures.

## 1. Introduction

The question of which diseases are harmful to human health and need to be treated is the foremost question in medicine. However, on the issue of thyroid cancer, we seem to have lost our way. In the past 30 years, thyroid cancer incidence rate has increased rapidly in most parts of the world and is more obvious in developed countries. In the 90s of the last century, the thyroid cancer incidence rate has increased slowly in Korea. The incidence rate of thyroid cancer increased sharply in 2000 and increased by 25% annually. In 2011, about 40568 new thyroid cancer patients were diagnosed, and the incidence rate of thyroid cancer was 15 times that of 1993 [1]. It is estimated that the standardized incidence rate of thyroid cancer in Korea is 8 times that of the United States and 10 times that of the world [2]. The incidence rate of thyroid cancer is almost attributable to the increase of papillary thyroid carcinoma, while the incidence rate of follicular thyroid carcinoma and medullary thyroid carcinoma is stable [2]. Park et al. investigated the incidence rate of thyroid cancer in South Korea in 1999–2008 and found that the proportion of papillary thyroid carcinoma increased continuously, and in 1999, it was 87.4%, rising to 97.4% in 2008, and the average tumor diameter was decreasing, 18 mm in 1999 and 8 mm in 2008 [3].Cho et al. summarized the clinicopathological and prognostic changes of thyroid cancer in South Korea in the past 40 years and found that the proportion of PTMC increased from 6.1% in 1962 to about 9% in 1990, to 54% in 2005, and to 43.1% in 2009 [4]. Despite the rapid increase in incidence rate, the standardized annual mortality of thyroid cancer has not increased significantly and has been maintained below 0.8/10 million. The mortality rate is 300–400 per year, accounting for 1% of the new thyroid cancer cases, and the 10-year survival rate is about 99.2% [5]. Despite the increased incidence of thyroid cancer, the mortality of thyroid cancer in the United States, South Korea, and many other countries has not increased significantly [6, 7]. The fact that the incidence of thyroid cancer is increasing rapidly while the mortality rate is relatively stable calls for attention.

The author believes that the overdiagnosis of thyroid cancer is the inevitable result of the rapid development of imaging diagnosis technology, which leads to a large number of thyroid nodules detected and the lack of understanding of the heterogeneity of malignant tumors, especially the biological behavior of PTMC. This article will objectively introduce the current situation, causes, clinical research progress, and coping strategies of PTMC overdiagnosis.

## 2. Analysis of the Incidence Rate of Thyroid Cancer

It is worth noting that the rapidly rising incidence rate of thyroid cancer is related to the large number of thyroid nodules. As we all know, in the past 30 years, great progress has been made in medical insurance systems, convenient healthcare system, and diagnostic imaging (ultrasound, CT, MRI, PET/CT, etc.). In 1999, the Korean government launched a cancer screening program for gastric cancer, breast cancer, cervical cancer, liver cancer, and colorectal cancer. Because of the simplicity and low cost of thyroid ultrasound, many women who are screening for breast cancer and cervical cancer also receive thyroid ultrasound. Research shows that, in 2009, about 13.2% of Korean adults (8.4% of men and 16.4% of women) were screened for thyroid cancer [8]. In 2011, thyroid ultrasound increased further, increasing to 15.8% in men and 31.3% in women [5]. In 2014, Udelsman and other National Cancer Project registration analyses calculated the incidence rate of thyroid cancer in the US in 1999–2009 and the incidence of thyroid cancer in various states of the United States. It was found that endocrinologist intensive, frequent cervical ultrasound examination and thyroid cancer detection rate were closely related [9]. Kim et al. pointed out that the wide application of high-resolution ultrasound is the only reason or main cause of the increase in thyroid cancer incidence rates [5]. Hoang et al. calculated the incidence rate of thyroid cancer from 1983 to 2009 and the amount of CT in 1993–2006. The linear regression analysis showed that CT was positively correlated with the detection rate of PTMC (*r* = 0.98, *P* < 0.01) [10]. Davis et al. believed that the development of medical imaging technology (such as high-resolution ultrasound) and diagnostic skills (such as experienced doctors conducting FNAB on 3 mm nodules under the guidance of ultrasound) led to the detection of a large number of asymptomatic subclinical thyroid cancer cases. However, this kind of tumor tends to grow in an inert state, and the progression of the disease that endangers the life of the host is highly unlikely. When the tumors were very small, they were examined and diagnosed. It is the result of “overdiagnosis” [6]. According to the data of Five Continents database established by the International Cancer Research Institute, Roman et al. pointed out that, during 1998–2007, about 70%–80% of American women with thyroid cancer had asymptomatic lesions; if there was no ultrasound or other imaging examination, these lesions would continue to hide in the body of patients, that is, the results of overdiagnosis of thyroid cancer. In addition, about 90% of South Korean women with thyroid cancer, 70–80% of Italian, French, and Australian women with thyroid cancer, and 50% of women with thyroid cancer in Japan, England, Scotland, and Northern European countries (Norway, Sweden, Denmark, and Finland) are all results of overdiagnosis of thyroid cancer [11]. Thyroid cancer patients, especially PTMC, increased significantly, but the specific mortality rate of thyroid cancer remained stable. The reason behind this phenomenon is the rapid development of medical diagnostic technology in recent decades. In fact, such seemingly positive diagnosis and treatment (including focusing on early detection, early diagnosis, and active and radical cure) do not necessarily reduce the mortality of these cancers. On the contrary, it may also increase the suffering of patients, plunge them into unnecessary fear of cancer, and significantly affect their subsequent quality of life.

## 3. Recognition of Biological Behavior of Papillary Thyroid Carcinoma

In fact, pathologists have realized that there are a large number of PTMC. These tumors grow slowly, have no clinical significance, and will not endanger patients' lives; they are also called “indolent cancers.” Due to differences in study population, slice thickness, diagnostic criteria, etc., the detection rate of thyroid cancer in autopsy fluctuates between 5% and 36%, generally below 10%, except for a few studies in Japan, Finland, Singapore, etc. [12]. In 2007, Japanese scholar Ito et al. found that 3.0–9.9 mm PTMC which can be found by ultrasound and the detection rate in autopsy is about 0.5%–5.3% [13]. From 1991 to 1995, Karamatsu et al. carried out routine thyroid ultrasonography on 15190 employees of Toyota Motor Company in Japan and found 59 thyroid cancer patients in total. The detection rate of thyroid cancer was about 0.39% [14]. Takebe et al. reported that Kumar Hospital in Japan used ultrasound and FNAB to carry out thyroid cancer screening work, and the detection rate of thyroid cancer in Japanese adult women over 30 years old was as high as 3.5% [15]. Chung et al. reported that 1401 breast cancer screening patients were screened by ultrasound and FNAB at the same time from 1997 to 1998. A total of 37 thyroid cancer patients were found, and the detection rate of thyroid cancer was 2.6% (37/1401) [16].

In view of the high detection rate of thyroid cancer detected by ultrasound and the detection rate of thyroid cancer in autopsy, the incidence rate of thyroid cancer in clinical setting was not high at that time. Therefore, Japanese scholar Miyauchi speculated that (1) most of the PTMC will remain dormant and will not progress or rarely progress and will not harm the health of the host; (2) most PTMC do not need surgical treatment; regular observation is enough; (3) during the observation period, some patients with PTMC may progress, and delayed surgical treatment will not have a significant impact on the prognosis of patients; (4) surgical treatment for all PTMC may do more harm than good. In 1993, Akira Miyauchi proposed the regular observation (no operation) for low-risk PTMC and obtained the consent of other surgeons and endocrinologists. Kumar Hospital launched the clinical study of regular observation of low-risk PTMC in 1993. In 1995, a similar study was initiated at the Tokyo Cancer Institute Hospital [13]. In 2003, Japanese scholar Ito first reported 162 cases of low risk (far away from the trachea and recurrent laryngeal nerve, no extrathyroidal invasion, FNA pathology did not indicate high-risk pathological subtypes). In addition, there were no suspicious or FNA confirmed metastatic lymph nodes in the lateral cervical region by ultrasonography. The average follow-up period was 4 years (46.5 ± 215 months), and the results showed that tumor volume did not increase in more than 70% of patients (≥2 mm), while only 1.2% of patients had new cervical lymph node metastasis, suggesting that low-risk PTMC rarely progressed to clinical dominant disease; for low-risk PTMC without clinical progress, observation strategy is a reasonable choice without affecting prognosis [17]. In 2010, Ito reported the observation results of 340 patients with low-risk PTMC. Thyroid ultrasound was performed 1–2 times per year. Two main indexes were observed: tumor volume and new lymph node metastasis. The observation period was 18–187 months (average 74 months). The follow-up results showed that tumor growth (>3 mm) in 5 and 10 years was 6.4% and 15.6%, respectively, and the proportion of new lymph node metastasis in 5 and 10 years was 14% and 3.4%, respectively [18]. In 2016, Oda et al. reported the observation results of 1179 cases of low-risk PTMC, with an average observation period of 47 months (12–116 months). It was found that only 2.3% (27 cases) of patients had tumor volume increase (≥3 mm), 0.5% (6 cases) of patients had new lymph node metastasis, and no patients had distant metastasis or died of thyroid cancer. However, the incidence of postoperative adverse events such as recurrent laryngeal nerve palsy, hypoparathyroidism, postoperative hematoma, and long-term need for thyroxine was significantly higher in the operation group than in the observation group [19]. Miyauchi shared the experience of clinical observation of more than 2000 low-risk PTMC patients in Kuma Hospital for 22 years. In 1235 patients in the observation group, 8% of them had tumor volume increase (≥3 mm), 3.8% had new cervical lymph node metastasis, in patients with mild tumor progression, delayed surgery can be successfully implemented, and no thyroid cancer specific death occurred in both groups. However, the incidence of temporary vocal cord paralysis and temporary and permanent hypoparathyroidism was 4.1% vs. 0.6% (*P* < 0.0001), 16.7% vs. 2.8% (*P* < 0.0001), and 1.6% vs. 0.08% (*P* < 0.0001) in the immediate operation group and the clinical observation group, respectively, indicating that the incidence of adverse events in the immediate operation group was significantly higher than that in the clinical observation group (Oda and Miyauchi et al., 2016). Hua SR et al. collected 1414 cases of PTMC patients in the general surgery department of Beijing Union Medical College Hospital and found that the overall lymph node metastasis rate of PTMC (≤0.5 cm) was not high; in particular, the lymph node metastasis rate was very low. It was considered that the observation strategy was a reasonable option, especially for female patients with a single focus aged ≥40 years [20]. Davies et al. analyzed the survival of 35663 cases of thyroid cancer (papillary carcinoma subtype, no extrathyroid invasion, no lymph node metastasis, and no distant metastasis) registered in SEER database from 1973 to 2005. Among them, 98.8% (35223 cases) received immediate standardized treatment, 1.2% (440 cases) did not receive standard treatment, the 20-year survival rate of the patients who received treatment and the patients who did not receive treatment was 99% and 97%, respectively, and there was no statistical significance between the two groups; Davies further pointed out that the 20-year tumor-specific mortality rate of the patients who did not receive treatment was about 2%–4%, while the incidence of permanent hypoparathyroidism and severe laryngeal function injury after thyroidectomy was about 3%–5% [21, 22]; therefore, the benefits and risks of patients need to be reconsidered. The evidence fully shows that most of the low-risk PTMC are inert growth tumors, which will not endanger the survival of the host, so clinical observation is a sensible option; surgical treatment cannot significantly improve the prognosis of low-risk PMC patients but will cost more medical resources and lead to more complications.

## 4. The Countermeasures for Overdiagnosis of PTMC

There is no doubt that the progress of medical imaging technology will contribute to the detection of early tumors and greatly improve the ability of clinicians to recognize, understand, and deal with diseases; however, once the core value of being people-oriented is forgotten, it may lead medicine in the wrong way of overdiagnosis. In the early 1950s, facing the rapid development of science and technology, Einstein, the master of science, pointed out with great concern that “this is an era of increasingly perfect means, but increasingly disordered goals,” which described the dilemma of the current PTMC overdiagnosis and overtreatment. Clearly, to get out of this dilemma, we should clear the goal of treatment, and “patient need” is our goal and direction.

### 4.1. Opposing Routine Thyroid Cancer Screening in Healthy People

At present, the research on thyroid cancer screening is not enough. As there was no significant difference in the prognosis between the low-risk PTMC clinical observation group and the treatment group, thyroid cancer screening in the asymptomatic population did not reduce tumor specific mortality; on the contrary, it leads to overdiagnosis and overtreatment. According to the “no harm principle” of medicine, in April 2015, South Korea first formulated a thyroid cancer screening guideline, which does not recommend routine thyroid cancer screening for the healthy population (Yi and Kim et al.). In May 2016, the new version of clinical practice guideline for the diagnosis and management of thyroid nodules, jointly issued by the American Association of Clinical Endocrinologists (AACE), American College of Endocrinology (ACE), and Associazione Medici Endocrinologi (AME), does not recommend the use of ultrasound for the screening of thyroid cancer in asymptomatic people [23]. In May 2017, the U.S. Preventive Services Task Force (USPSTF) believed that there was currently moderate- or high-quality evidence that the screening of thyroid cancer in asymptomatic population has no net benefit or damage greater than the benefit and issued guidelines for thyroid cancer screening, with the recommended level of *D*; that is, it is against the screening of thyroid cancer in asymptomatic population by neck palpation, neck ultrasonography, or other means [24]. In my opinion, this is not to say that ultrasound screening for thyroid cancer has no value. On the contrary, we should reexamine the efficiency and limitations of screening, so that thyroid cancer screening can better serve the clinical necessity.

### 4.2. Strict Control of Indications of Thyroid Nodule Biopsy

The American Thyroid Association recognizes that the vast majority of thyroid cancers are inert tumors that develop slowly and that early detection and treatment do not really benefit patients. As a feedback to the overdiagnosis, the guideline for the diagnosis and treatment of thyroid nodule and differentiated thyroid cancer published by American Thyroid Association in 2015, the indications for FNAB biopsy are more stringent: generally speaking, FNAB is recommended for thyroid nodules with high risk and diameter ≥1 cm, while FNAB is not recommended for thyroid nodules with diameter smaller than 1 cm, even if ultrasound indicates that the nodule has malignant signs. FNAB is not recommended for evaluation and implementation, which means that the vast majority of PTMC will not be confirmed by pathology [25]. For example, for thyroid nodules less than 1 cm, even if ultrasound indicates suspicious malignancy (such as unclear border, microcalcification, vertical and horizontal imbalance, etc.), ATA guidelines in 2015 also recommend strict ultrasound follow-up observation instead of immediate FNAB examination. In 2016, the new version of clinical practice guideline for the diagnosis and management of thyroid nodules issued by the American Association of Clinical Endocrinologists (AACE), American College of Endocrinology (ACE), and Associazione Medici Endocrinologi (AME) also recommended FNAB only for thyroid nodules with high malignant possibility and diameter ≥ 1 cm, while nodules with a major diameter <5 mm should be monitored, rather than biopsied, with US, irrespective of their sonographic appearance; for nodules with a major diameter 5–10 mm that are associated with suspicious US signs (high US risk thyroid lesions), consider either FNA sampling or watchful waiting on the basis of the clinical setting and patient preference [23].

### 4.3. Readjustment of the Pathological Standard of Some Thyroid Cancer

On March 8–9, 2008, the National Cancer Institute (NCI) held a special meeting to discuss the “overdiagnosis” and “overtreatment” of cancer, pointing out that if tumors do not produce symptoms or cause death, the diagnosis and treatment of these tumors belong to overdiagnosis and overtreatment. Oncologists agree that, in order to avoid being confused with cancer that endangers the lives of patients, inert cancer that does not harm the health of the host should be separated from cancer in the traditional sense and renamed as “inert lesion of epithelial origin” [26]. Nikiforov et al. followed up 109 patients with encapsulated follicular variant of papillary thyroid carcinoma (EFVPTC) for 10–26 years (mean 14.4 years). No recurrence, metastasis, or death occurred. After repeated discussion and consultation, thyroid pathologists decided to rename it as “noninvasive follicular thyroid tumor with papillary like nuclear features (NIFTP)”, which no longer belongs to the category of thyroid carcinoma [27]. It is estimated that this measure is expected to reduce about 20% of patients with papillary thyroid cancer, avoiding unnecessary surgery, medical costs, and psychological burden. In April 2017, the American Thyroid Association issued guidelines which confirmed the change of “wrapped follicular papillary thyroid cancer” to “noninvasive follicular thyroid tumor with papillary nuclear characteristics.” However, in view of the moderate level of evidence quality and weak recommendation, prospective research is needed [25]. Xu et al. found that NIFTP of ≥4 cm (4 cm–8 cm) can achieve satisfactory results only by surgical treatment (including adenoidectomy, etc.), and there is no need for radioiodine treatment after operation, and the risk of recurrence is very low [28].

### 4.4. Gradually Accepting the Observation Strategy of Thyroid Cancer in Clinical Guidelines

For more than 20 years, research in Kuma Hospital and Tokyo Cancer Research Institute has proved that the clinical observation of low-risk PTMC is safe and effective and can be the first option for patients with low-risk PTMC. According to the Japanese thyroid cancer diagnosis and treatment guidelines published by the Japanese Society of Endocrinologists and the Japanese Society of Thyroid Surgery in 2010, for patients with low-risk (no cervical lymphadenopathy, no extrathyroid invasion, and no distant metastasis) PTMC, the clinical observation and follow-up is also one of the treatment plans [29]. In 2016, for the first time, surgical treatment is generally recommended, and clinical observation can also be carried out for patients with low risk who are confined to the gland and have no lymph node metastasis [25]. According to the “Chinese expert consensus and guidelines for the diagnosis and treatment of papillary thyroid microcarcinoma” issued by the Chinese Association of Thyroid Oncology in 2016, the treatment scheme of intraglandular PTMC (especially the diameter ≤5 mm) should be determined after comprehensive analysis of clinical stage and risk assessment and full communication with patients and their families. Close observation can be recommended for patients who meet all the following conditions: ① nonpathological high-risk subtype; ② tumor diameter ≤5 mm; ③ tumor not close to the thyroid capsule and there is no invasion of surrounding tissues; ④ no evidence of lymph node or distant metastasis; ⑤ no family history of thyroid cancer; ⑥ no history of neck radiation exposure in youth or childhood; ⑦ patients having little psychological pressure and can actively cooperate. Surgical treatment should be considered in the following situations during the observation: ① tumor diameter increases more than 3 mm; ② clinical lymph node metastasis is found; ③ patients change their wishes and require surgery [30].

## 5. Conclusion

The incidence rate of thyroid cancer increases at 20.1% per year. The reason is the overdiagnosis of thyroid cancer. 2400 years ago, Hippocrates, the father of medicine, warned us, “never do too much on patients.” We need to elucidate strategies to mitigate overdiagnosis, improve clinician and patient communication about it, and prevent overtreatment. These goals will require concerted efforts from medical societies, public agencies, and healthcare foundations.

## Data Availability

The data used to support the findings of this study are cited as references.
